# Perceived stress and severity of male etomidate use disorder in China: a moderated mediation model of anxiety symptoms severity and emotion regulation difficulties

**DOI:** 10.3389/fpsyg.2026.1778758

**Published:** 2026-05-20

**Authors:** Xingmin Wang, Juan Le, Ying Tang, Qiuping Huang, Jing Qi, Xinxin Chen, Lishun Zhao, Lin Zhao, Zhenjiang Liao, Hongxian Shen

**Affiliations:** 1 Department of Psychiatry, National Clinical Research Center for Mental Disorders, The Second Xiangya Hospital of Central South University, Changsha, Hunan, China; 2 Department of Psychology, School of Humanities and Management, Hunan University of Chinese Medicine, Changsha, Hunan, China; 3 Department of Psychiatry, Brain Hospital of Hunan Province, Changsha, Hunan, China; 4 Hunan Provincial Lushan Compulsory Isolation Detoxification Center, Changsha, Hunan, China

**Keywords:** anxiety symptoms severity, difficulties in emotion regulation, etomidate use disorder, perceived stress, moderated mediation

## Abstract

**Background:**

Non-medical use of etomidate has emerged as a novel psychoactive substance (NPS) threat in China. Although stress-related mechanisms have been widely implicated in substance use disorders, evidence specific to etomidate use disorder (EUD) remains limited. This study examined whether anxiety symptoms (AS) severity mediates the association between perceived stress and EUD severity, and whether difficulties in emotion regulation strengthen the stress–AS severity link.

**Methods:**

A total of 556 male patients diagnosed with EUD were recruited from drug rehabilitation centers in Hunan Province, China (M_*age*_ = 22.19, SD = 5.96). Participants completed measures of perceived stress (CPSS), AS severity (BAI), and difficulties in emotion regulation (DERS). EUD severity was indexed by DSM-5 criterion count (0–11). A moderated mediation model was tested using PROCESS (Model 7) with bias-corrected bootstrapping (5,000 resamples), controlling for age, educational attainment, only-child status, single-parent family status, and residence (rural vs. urban).

**Results:**

Perceived stress was positively associated with EUD severity. AS severity partially mediated the association between perceived stress and EUD severity. Moreover, DERS moderated the perceived stress–AS severity path, such that perceived stress was more strongly related to AS severity at higher levels of emotion regulation difficulties. Consistently, the indirect effect of perceived stress on EUD severity through AS severity was stronger among individuals reporting greater emotion regulation difficulties.

**Conclusion:**

These findings suggest that AS severity serves as a potential emotional correlate in the relationship between perceived stress and EUD severity, particularly among individuals with substantial emotion regulation difficulties. Individuals with greater deficits in emotion regulation exhibited a stronger indirect effect of stress on EUD severity via AS severity. This highlights the clinical importance of targeting emotion regulation in interventions for EUD, as improving emotional coping strategies may mitigate the impact of stress. Future longitudinal studies are needed to clarify the causal direction of these associations and investigate how changes in emotion regulation over time influence EUD progression.

## 1 Introduction

Etomidate is an ultrashort-acting, non-barbiturate intravenous sedative widely used for procedural sedation and the induction of general anesthesia (DrugBank, 2025; Gao et al., 2025). However, non-medical use of etomidate has emerged as a novel psychoactive substance (NPS), particularly in China and South Korea in recent years (Liu et al., 2025; Uhm et al., 2024). Increasing evidence indicates that etomidate misuse is associated with serious adverse consequences, including neurotoxicity, aggression, adrenal insufficiency, and endocrine dysfunction. Despite growing clinical concern, empirical research on the psychological mechanisms underlying etomidate use disorder (EUD) remains extremely limited.

A large body of addiction research has identified stress, negative affect, impulsivity, and emotion regulation difficulties as key contributors to substance use disorders (SUDs) (Sinha, 2024; Stellern et al., 2023). However, whether and how these established mechanisms apply to EUD, an emerging and pharmacologically distinct substance, has not been systematically examined. Addressing this gap is essential for developing targeted prevention and intervention strategies for individuals with EUD.

### 1.1 Perceived stress and etomidate use disorder

Perceived stress is defined as “feelings or thoughts that an individual has about how much stress they are under at a given point in time or over a given time period” (Phillips, 2013). Extensive evidence demonstrates a robust association between stress exposure and substance use across different substances and populations. Neurobiological research suggests that chronic stress can dysregulate the corticotropin-releasing factor–hypothalamic–pituitary–adrenal (CRF/HPA) axis and alter cortico-striatal-limbic circuits involved in reward processing, motivation, and self-control (Kirsch et al., 2025; MacLean et al., 2019; Sinha, 2008). These stress-related neuro-adaptations may increase vulnerability to compulsive substance use. From a psychological perspective, substance use has been conceptualized as a maladaptive coping strategy for alleviating stress-related distress, anxiety, and negative affect (Khantzian, 1985; Sinha, 2001). According to the adaptive stress response framework, repeated exposure to stress, trauma, and adversity may interact with escalating substance use and withdrawal-related distress, ultimately amplifying craving and increasing the likelihood of relapse (Sinha, 2024). Although perceived stress has been identified as a key vulnerability factor in various SUDs, its role in EUD has rarely been examined.

Based on this literature, we hypothesized that higher levels of perceived stress would be positively associated with greater EUD severity (Hypothesis 1).

### 1.2 The mediating role of anxiety symptoms severity

Anxiety disorders are among the most prevalent mental health conditions worldwide and frequently co-occur with substance use disorders (Castaldelli-Maia and Bhugra, 2022). Substantial evidence indicates that perceived stress is a proximal risk factor for anxiety symptoms (AS) severity, as chronic or intense stress activates neuroendocrine systems and disrupts emotion-related brain regions such as the amygdala and prefrontal cortex, impairing fear regulation and increasing anxiety vulnerability (Buckner et al., 2021; Ipser et al., 2015).

Anxiety symptom severity, in turn, has been consistently linked to substance use through multiple pathways. Individuals experiencing heightened AS severity may engage in substance use to reduce aversive emotional and physiological states (negative reinforcement) or to facilitate social functioning and confidence (positive reinforcement) (Buckner et al., 2021; Rieselbach et al., 2023). Cognitive impairments associated with AS severity, including biased threat appraisal and impaired decision-making, may further increase susceptibility to compulsive substance use (Akiki et al., 2025; Nyberg et al., 2021). Pharmacologically, etomidate primarily exerts its rapid sedative and anxiolytic effects via modulation of the gamma-aminobutyric acid (GABA) system, which may directly ameliorate anxiety-related hyperarousal states (Heldt and Ressler, 2007). Moreover, existing research indicates that AS severity represents a significant predictor of sedative-hypnotic drug misuse, suggesting that individuals with higher AS severity are more likely to employ such medications as a coping strategy for negative affective distress (Starcevic et al., 2016; Sun et al., 2024).

Integrating these perspectives, AS severity may represent a key emotional mechanism through which perceived stress is associated with EUD severity (Fang et al., 2023). Therefore, we hypothesized that AS severity would mediate the relationship between perceived stress and EUD (Hypothesis 2).

### 1.3 The moderating role of difficulties in emotion regulation

Emotion regulation refers to processes by which individuals monitor, evaluate, and modify emotional responses to achieve adaptive functioning (Gross, 2002). Individuals differ substantially in their capacity to regulate emotions under stress. Difficulties in emotion regulation have been associated with heightened cortisol responses, prefrontal regulatory dysfunction, and increased repetitive negative thinking (Gross, 2002; Langer et al., 2022; Mansueto et al., 2022), all of which may increase vulnerability to AS severity and substance use (Mansueto et al., 2024b).

From a stress–coping perspective, individuals with greater emotion regulation difficulties may be less able to flexibly deploy adaptive coping strategies when confronted with stressors. As a result, perceived stress may be more likely to translate into heightened AS severity among these individuals, whereas those with better emotion regulation capacities may be able to buffer or downregulate stress-related emotional responses.

Accordingly, we hypothesized that difficulties in emotion regulation would moderate the association between perceived stress and AS severity, such that this association would be stronger at higher levels of emotion regulation difficulties (Hypothesis 3).

### 1.4 Aims and hypotheses

**FIGURE 1:**
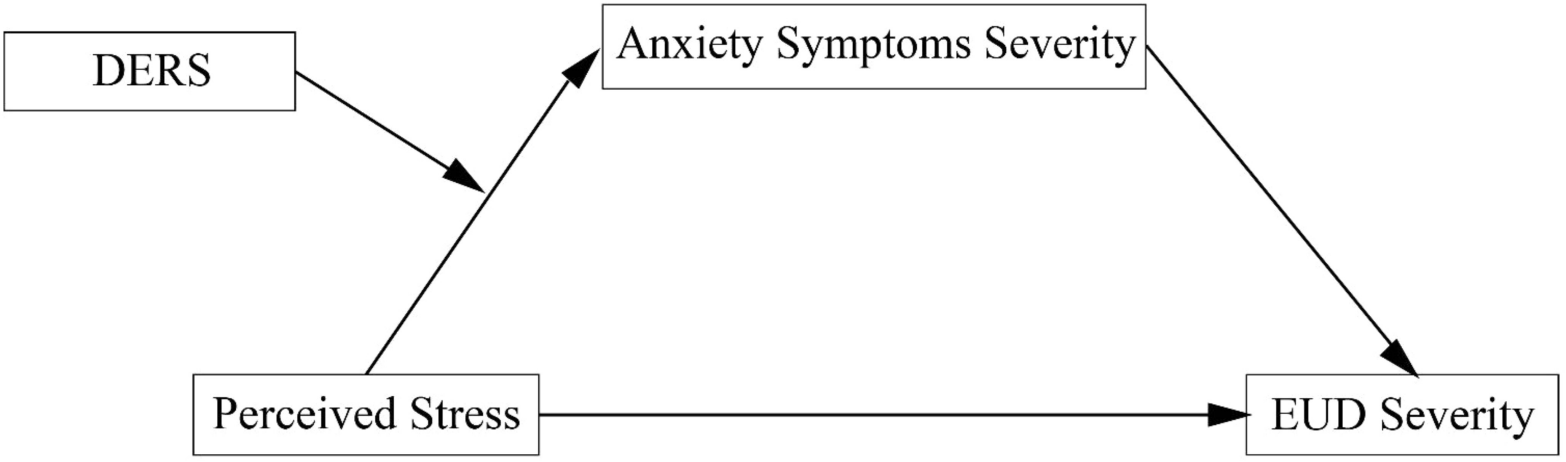
The theoretical model. DERS, Difficulties in Emotion Regulation; EUD, etomidate use disorder.

The present study aimed to clarify the psychological mechanisms linking perceived stress to EUD severity by testing a moderated mediation model (Figure 1). Specifically, we examined whether AS severity mediates the association between perceived stress and EUD severity, and whether difficulties in emotion regulation moderate the stress–AS severity pathway.

The study’s hypotheses are:

**H1:** Perceived stress is positively associated with EUD severity.

**H2:** AS severity mediates the association between perceived stress and EUD severity.

**H3:** Difficulties in emotion regulation moderate the association between perceived stress and AS severity, such that the association is stronger at higher levels of emotion regulation difficulties.

## 2 Materials and methods

### 2.1 Participants

Participants were recruited between June 2023 and June 2025 from drug rehabilitation center in Hunan Province, China. All participants were male. Participants in this study were recruited from a drug rehabilitation facility that caters to both individual seeking voluntary treatment and those referred via the judicial system. Eligibility for recruitment was determined by standardized hair toxicology screening, with the key criterion being a history of etomidate use within the preceding 6 months.

Diagnosis of EUD was established by trained psychiatrists according to the Diagnostic and Statistical Manual of Mental Disorders, Fifth Edition (DSM-5) (Hasin et al., 2013). Participants were excluded if they reported (a) polydrug use, (b) serious physical illness requiring ongoing medical treatment, or (c) current or past major psychiatric disorders, including schizophrenia spectrum disorders, anxiety disorder, and other psychotic conditions. A total of 567 individuals were initially screened. Of these, four were excluded due to a prior diagnosis of psychiatric disorders and seven declined participation, resulting in a final analytical sample of 556 participants. This study sample was a mixed cohort, comprising individuals who voluntarily sought rehabilitation treatment and those referred through the judicial system.

The mean age was 22.19 years (±5.96 SD). Among them, 50.9% of participants had middle school education, 24.6% were one-child family, 27.7% came from single - parent families, and 96.8% lived in urban areas. All the details can be found in Table 1.

**TABLE 1:** Demographic characteristics of the participants.

Variables	*N*	%
Total	556	100.0
Education level		
Primary school and below	138	24.8
Middle school	283	50.9
High/vocational school	103	18.5
Bachelor’s degree or higher	32	5.8
Whether an only child		
Yes	137	24.6
No	419	75.4
Whether a single-parent family		
Yes	154	27.7
No	402	72.3
Residence		
Rural	18	3.2
Urban	538	96.8

### 2.2 Measures

#### 2.2.1 The Chinese Perceived Stress Scales

Perceived stress was assessed using the Chinese Perceived Stress Scale (CPSS), developed by Yang et al. (2003) to reflect stress experiences within the Chinese cultural context. The CPSS consists of 14 items measuring the degree to which individuals perceived their lives as stressful over the past month, ranging from 0 (never) to 4 (very often). Total scores range from 0 to 56, with higher scores indicating greater perceived stress. Previous studies have reported acceptable internal consistency for the CPSS (Cronbach’s α = 0.71–0.78) (Li et al., 2021). In the present study, the CPSS demonstrated good internal consistency (Cronbach’s α = 0.74).

#### 2.2.2 The Beck Anxiety Inventory

Anxiety symptoms (AS) were measured using the Beck Anxiety Inventory (BAI), a widely used self-report scale designed to assess AS severity while minimizing overlap with depressive symptoms (Beck et al., 1988). The BAI comprises 21 items assessing common somatic and cognitive AS experienced over the past week. Items are rated on a four-point Likert scale, yielding total scores ranging from 0 to 63, with higher scores indicating greater AS severity. The BAI has demonstrated excellent internal consistency in previous studies (Cronbach’s α = 0.90–0.94) (Julian, 2011). In the present study, the BAI showed high internal consistency (Cronbach’s α = 0.94).

#### 2.2.3 The Difficulties in Emotion Regulation Scale

Difficulties in emotion regulation were assessed using the Difficulties in Emotion Regulation Scale (DERS), a 36-item self-report instrument designed to evaluate multiple dimensions of emotion regulation difficulties (Gratz and Roemer, 2004). Items are rated on a five-point Likert scale ranging from 1 (almost never) to 5 (almost always). The DERS yields a total score and six subscale scores, with higher scores indicating greater difficulties in emotion regulation. The scale has demonstrated strong psychometric properties, with an overall Cronbach’s α of approximately 0.90 (Li et al., 2018). In the present study, the DERS demonstrated excellent internal consistency (Cronbach’s α = 0.94).

#### 2.2.4 Etomidate use disorder severity

Etomidate use disorder severity was indexed using the DSM-5 diagnostic criteria (Hasin et al., 2013). Trained psychiatrists assessed the presence of each of the 11 DSM-5 criteria for substance use disorders through structured clinical interviews. EUD severity was operationalized as the total number of criteria endorsed within the past 12 months, with possible scores ranging from 0 to 11. Higher scores indicate greater severity of EUD.

#### 2.2.5 Recent craving score

The Visual Analog Scale (VAS) was administered to assess substance craving severity. The standardized instruction provided to participants was: *“Please imagine that you are in an unconstrained environment and have observed another individual using etomidate. Rate the intensity of your current craving, with 0 indicating no craving and 10 indicating extremely strong craving.”* Single-item VAS measures are widely used in addiction research to assess momentary craving intensity and have been shown to exhibit adequate validity and test-retest reliability (Boyett et al., 2021; Dunbar et al., 2010; Goodyear and Haass-Koffler, 2020; Miranda et al., 2019). In the present study, higher VAS scores corresponded to greater levels of subjective craving. For the sensitivity analysis, this craving measure was designated as an alternative outcome variable, rather than serving as a direct indicator of EUD severity.

### 2.3 Procedure

All participants were interviewed by trained psychiatrists. Demographic information and self-report questionnaires were administered under the supervision of a physician. The authors assert that all procedures contributing to this work comply with the ethical standards of the relevant national and institutional committees on human experimentation and with the Helsinki Declaration of 1975, as revised in 2013. All procedures involving research study participants were approved by the Ethics Committee of the Second Xiangya Hospital, Central South University (No. 2023K003). Written informed consent was obtained from all participants prior to data collection. For participants under 18 years of age, written consent was additionally obtained from their parents or legal guardians.

### 2.4 Data analysis

Statistical analyses were conducted using SPSS version 26.0 and the PROCESS macro for SPSS. Prior to hypothesis testing, Harman’s single-factor test was used to examine potential common method bias. Descriptive statistics and Pearson correlation analyses were then performed for all study variables. Missing values were handled using multiple imputation under the assumption of missing at random. The proportion of missing data for each variable is presented in Supplementary Table 1. Despite the low overall missing rate (<1%), multiple imputation was employed as a conservative strategy to ensure data processing consistency and minimize potential biases. Multiple imputation was performed using the fully conditional specification method in SPSS. The imputation model included all variables used in the primary analyses, including demographic variables, perceived stress, AS severity, emotion regulation difficulties, and EUD severity. Five imputed datasets were generated. Moderated mediation analyses were conducted separately for each imputed dataset, and parameter estimates and standard errors were pooled using Rubin’s rules.

Mediation analyses were conducted using PROCESS Model 4 to examine whether AS severity mediated the association between perceived stress and EUD severity. Moderated mediation analyses were performed using PROCESS Model 7 to test whether difficulties in emotion regulation moderated the first-stage path from perceived stress to AS severity. All models adjusted for the demographic variables listed in Table 1 as covariates. Age was treated as continuous variables, whereas the coding schemes for categorical variables are specified in Supplementary Tables 2, 3. Sensitivity analyses used the VAS score as an alternative measure of outcome variable to assess model robustness.

All continuous variables were standardized prior to analysis. Ordinary least squares regression was used, and indirect effects were estimated using bias-corrected bootstrapping with 5,000 resamples. Effects were considered statistically significant if the 95% bootstrap confidence interval did not include zero. Conditional indirect effects were examined at low (M − 1 SD), mean (M), and high (M + 1 SD) levels of difficulties in emotion regulation.

## 3 Results

### 3.1 Common method bias

Harman’s single-factor test was conducted to examine potential common method bias. The unrotated exploratory factor analysis revealed 17 factors with eigenvalues greater than 1, and the first factor accounted for 27.98% of the total variance, which is below the commonly used threshold of 40% (Podsakoff et al., 2003). These results suggest that common method bias is unlikely to be a serious concern in the present study.

### 3.2 Descriptive analyses

Table 2 presents the means, standard deviations, and Pearson correlations among all study variables. Perceived stress was positively correlated with AS severity (*r* = 0.45, *p* < 0.01), difficulties in emotion regulation (*r* = 0.44, *p* < 0.01), and EUD severity (*r* = 0.23, *p* < 0.01). AS severity was also positively associated with difficulties in emotion regulation (*r* = 0.49, *p* < 0.01) and EUD severity (*r* = 0.34, *p* < 0.01). In addition, difficulties in emotion regulation were positively correlated with EUD severity (*r* = 0.19, *p* < 0.01).

**TABLE 2:** Results of descriptive statistics and correlation analysis.

Variables	1	2	3	4	M ± SD
1. Perceived stress	1	–	–	–	28.53 ± 7.26
2. AS severity	0.45**	1	–	–	8.23 ± 9.38
3. DERS	0.44**	0.49**	1	–	98.62 ± 10.54
4. EUD severity	0.23**	0.34**	0.19**	1	7.15 ± 3.12

*N* = 556;

**p < 0.01. DERS, Difficulties in Emotion Regulation; EUD, etomidate use disorder; AS severity, anxiety symptoms severity.

Overall, the observed correlations were in the expected directions and provided preliminary support for the hypothesized associations among perceived stress, AS severity, difficulties in emotion regulation, and EUD severity.

To clarify the clinical significance of AS severity in the present study, we further analyzed the frequency distribution of participants across established clinical cut-off scores for the BAI. Specifically, 62.1% of participants were categorized into the minimal anxiety symptoms range (0–7), 20.7% into the mild range (8–15), 10.6% into the moderate range (16–25), and 6.7% into the severe range (26–63). Notably, 17.3% of the sample demonstrated moderate-to-severe anxiety symptoms, indicating the presence of a clinically significant subgroup.

### 3.3 Mediation analysis

Mediation analyses were conducted using PROCESS Model 4 to examine whether AS severity mediated the association between perceived stress and EUD severity. All continuous variables were standardized prior to analysis, with the demographic variables listed in Table 1 included as covariates.

Results indicated that perceived stress was positively associated with EUD severity (β = 0.25, *p* < 0.001), indicating a significant total effect. Perceived stress was also positively associated with AS severity (β = 0.44, *p* < 0.001). In turn, AS severity was positively associated with EUD severity when controlling for perceived stress (β = 0.31, *p* < 0.001). Bootstrap analyses revealed a significant indirect effect of perceived stress on EUD severity through AS severity (standardized indirect effect = 0.13), with a 95% bias-corrected confidence interval of [0.10, 0.17]. Because the direct effect of perceived stress on EUD severity remained significant after accounting for AS severity, the mediation was partial. These results were consistent with Hypothesis 2.

### 3.4 Moderated mediation analysis

A moderated mediation analysis was conducted using PROCESS Model 7 to test whether difficulties in emotion regulation moderated the first-stage path from perceived stress to AS severity.

Results showed that the interaction between perceived stress and difficulties in emotion regulation was a significant positive predictor of AS severity (β = 0.098, *t* = 3.07, *p* = 0.002), indicating that difficulties in emotion regulation moderated the association between perceived stress and AS severity. Detailed results are presented in Supplementary Tables 4, 5.

**FIGURE 2:**
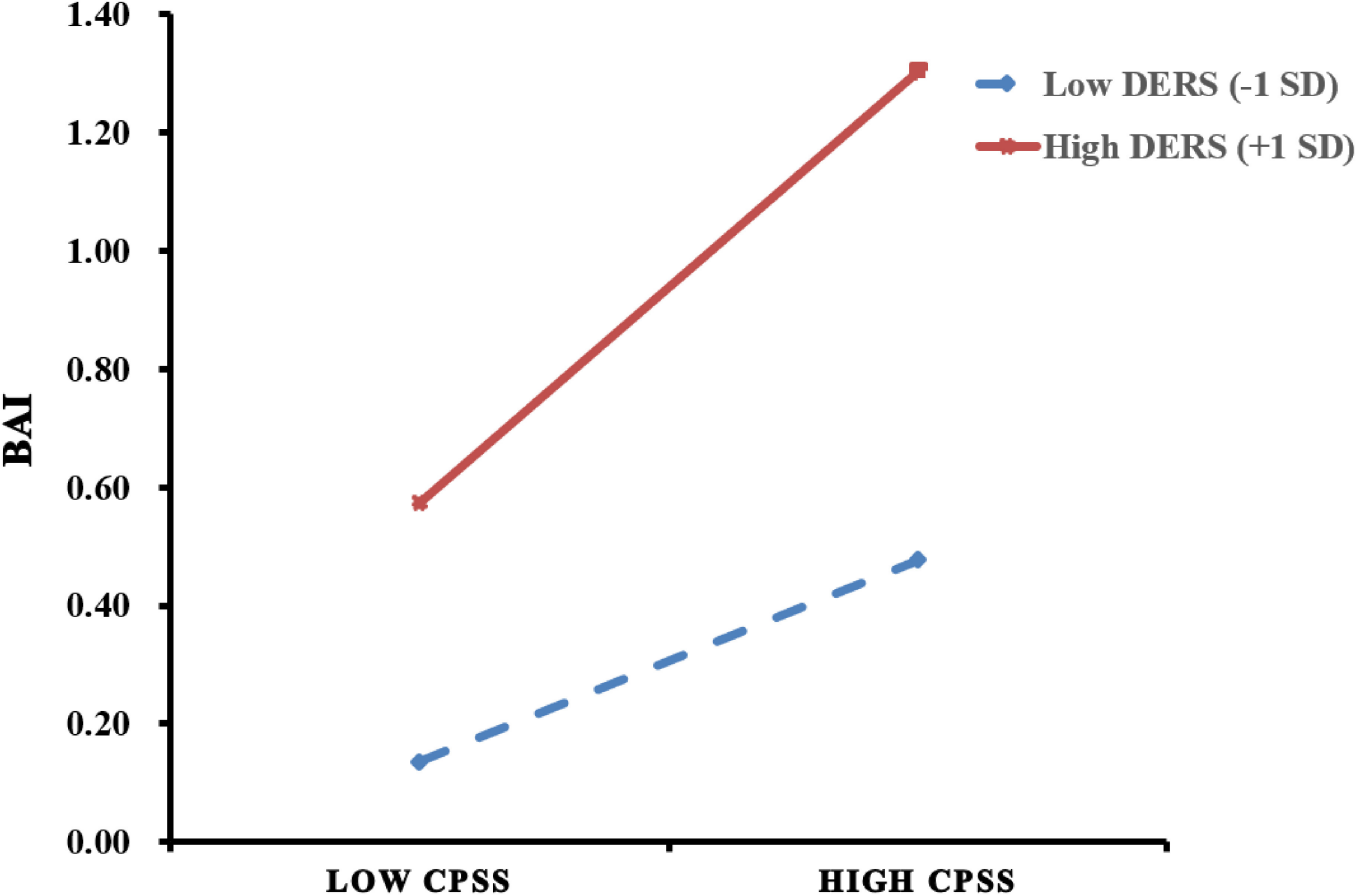
Moderating effect of difficulties in emotion regulation (DERS) on the association between perceived stress (CPSS) and anxiety symptom severity (BAI). Perceived stress was positively associated with anxiety symptom severity at both high (+1 SD; red line) and low (–1 SD; blue dashed line) levels of DERS, with a stronger association observed among individuals with higher DERS. The x-axis represents CPSS scores (low to high), and the y-axis represents BAI scores.

To further interpret this interaction, simple slope analyses were conducted at low (M − 1 SD) and high (M + 1 SD) levels of difficulties in emotion regulation. When difficulties in emotion regulation were low, perceived stress was positively associated with AS severity (β = 0.170, SE = 0.055, *p* < 0.001). When difficulties in emotion regulation were high, the association between perceived stress and AS severity was significantly stronger (β = 0.366, SE = 0.048, *p* < 0.001). These interaction effects are illustrated in Figure 2.

Notably, the moderated mediation index was 0.030, with a 95% bootstrap confidence interval of [0.008, 0.055] that excluded zero, confirming the presence of a significant moderated mediation effect. Conditional indirect effects of perceived stress on EUD severity via AS severity at different levels of difficulties in emotion regulation are presented in Table 3. The indirect effect was smallest at low levels of difficulties in emotion regulation and increased progressively at mean and high levels, indicating that greater emotion regulation difficulties amplified the indirect association between perceived stress and EUD severity through AS severity.

**TABLE 3:** Mediating effects of perceived stress at different levels of DERS.

DERS	Indirect effect	Boot standard deviation	Bootstrap low limit	Bootstrap upper limit
M−SD	0.052***	0.015	0.022	0.082
M	0.082***	0.015	0.053	0.112
M+SD	0.112***	0.022	0.068	0.156

Reported coefficients correspond to standardized effects (β); indirect effects were estimated using bias-corrected bootstrap confidence intervals based on 5,000 resamples. M, mean, SD, standard deviation.

***p < 0.001. DERS, Difficulties in Emotion Regulation.

Given the relatively low overall anxiety level in the sample, we caution that the moderated mediation results primarily reflect patterns within the full sample. Additional subgroup analyses stratified by AS were conducted to investigate whether the associations differed among participants with clinically elevated anxiety (moderate-to-severe anxiety symptoms). These subgroup analyses revealed that the indirect effect did not reach statistical significance in the clinical AS subgroup, and the moderated mediation index was non-significant in both subgroups. This further highlights the limitation pertaining to insufficient statistical power for detecting such effects in smaller subgroups with clinically elevated AS. Detailed results are presented in Supplementary Tables 6–9.

### 3.5 Sensitivity analysis

Sensitivity analysis was performed by substituting the outcome variable with recent craving scores. Pooled estimates derived from the five multiply imputed datasets showed that perceived stress was significantly positively associated with AS severity (β = 0.44, *p* < 0.001), which in turn was significantly positively associated with recent craving scores (β = 0.16, *p* < 0.001). The indirect effect of perceived stress on recent craving scores through AS severity was significant (β = 0.07, 95% CI [0.03, 0.11]), supporting partial mediation. Furthermore, the moderated mediation model revealed a significant interaction between perceived stress and emotion regulation difficulties (β = 0.098, *p* = 0.002).

## 4 Discussion

The present study examined the psychological mechanisms linking perceived stress to EUD severity by testing a moderated mediation model among male patients with EUD. Three main findings emerged. First, perceived stress was positively associated with EUD severity. Second, AS severity partially mediated this association, suggesting that perceived stress may be linked to EUD severity both directly and indirectly through AS severity. Third, difficulties in emotion regulation moderated the association between perceived stress and AS severity, such that the indirect effect of perceived stress on EUD severity through AS severity was stronger among individuals with greater emotion regulation difficulties. Together, these findings provide an integrated account of how stress-related emotional processes may be associated with EUD severity.

### 4.1 The direct effect between perceived stress and EUD

The present findings demonstrated a significant positive association between perceived stress and EUD severity, supporting Hypothesis 1. This result is consistent with prior research identifying perceived stress as a key vulnerability factor for substance use disorders (Khantzian, 1985; Sinha, 2001, 2024). From a stress-adaptation perspective, repeated exposure to stress may be associated with dysregulation of stress-responsive neurobiological systems, including the CRF/HPA axis, as well as alterations in reward- and control-related brain circuits (Sinha et al., 2006, 2007). Such stress-related adaptations may increase susceptibility to compulsive substance use and greater disorder severity.

Importantly, the association observed in the present study does not imply causality. Rather, the findings suggest that higher levels of perceived stress are associated with greater EUD severity in this clinical population. Identifying and addressing stress-related experiences may therefore represent a clinically relevant target in the assessment and management of individuals with EUD.

### 4.2 The mediating effect of anxiety symptoms severity

Anxiety symptom severity was found to partially mediate the association between perceived stress and EUD severity, consistent with Hypothesis 2. This finding suggests that perceived stress may be linked to greater EUD severity in part through heightened AS.

Notably, the sample demonstrated moderate-to-severe EUD, with a mean DSM-5 symptom count of 7.15, yet overall anxiety symptom levels remained low. Etomidate, a commonly used intravenous anesthetic in clinical settings, may increase the likelihood that patients with EUD rely on its abuse to alleviate AS, owing to the drug’s rapid sedative properties. However, this AS relief is transient: when underlying stressors remain unaddressed, AS recurs, driving patients to re-engage in etomidate abuse. Furthermore, DSM-5 primarily assess behavioral dyscontrol, compulsive use, tolerance, withdrawal, and functional impairment, rather than current emotional distress.

In the first half of the mediation model, perceived stress positively predicts AS severity. Neurobiologically, chronic stress activates the HPA axis, elevating cortisol and disrupting regions like the amygdala and prefrontal cortex, impairing fear modulation and heightening AS severity risk (Fang et al., 2023). Psychologically, Lazarus’s Stress and Coping Theory posits that stress is a dynamic cognitive process involving the interaction between individuals and their environment (Acoba, 2024; Folkman et al., 1986). Higher perceived stress is closely associated with threat appraisals and vulnerability perceptions, fostering AS severity. Similarly, Beck’s Generic Cognitive Model links AS to threat-oriented beliefs, intensified by perceived stress (Beck and Haigh, 2014). Based on these theories, perceived stress may be associated with AS in EUD patients by promoting threatening interpretations of stressful events and fostering perceptions of vulnerability.

The second half shows AS severity positively predicts EUD. This aligns with findings from the biopsychosocial model of anxiety-substance use disorder comorbidity proposed by Buckner et al. (2021). Individuals experiencing elevated anxiety symptoms may increase their risk of EUD through mechanisms such as managing negative emotions/physiological arousal, promoting social interaction, avoiding evaluation, and post-event processing (Buckner et al., 2021). The observed patterns in the current study are correlational, and it should be emphasized that only a minority of participants presented clinically elevated anxiety. Therefore, while coping-motivated EUD use represents a plausible mechanism mediating the relationship between anxiety symptom (AS) severity and EUD severity, the present data do not confirm that EUD serves definitively as an AS-alleviating strategy. The interpretation of EUD as a potential AS-alleviating or self-medication mechanism should thus be advanced with caution. Future research employing larger clinical samples and stratified study designs is warranted to more conclusively investigate the potential self-medication mechanism.

Beyond emotional symptoms, functional impairments associated with EUD span multiple domains, including impaired control over drug use, drug craving, and reckless drug-taking behaviors. As core indicators of addiction severity, these features may be governed by mechanisms partially independent of concurrent AS severity. Within the framework of multidimensional addiction theory, emotional distress represents only one dimension of functional impairment. Other potential mechanisms warrant further investigation.

### 4.3 The moderating effect of DERS

The present study further demonstrated that difficulties in emotion regulation moderated the association between perceived stress and AS severity, consistent with Hypothesis 3. Specifically, perceived stress was more strongly associated with AS severity among individuals reporting greater emotion regulation difficulties (Mansueto et al., 2022; Wells and Cartwright-Hatton, 2004).

This finding highlights emotion regulation capacity as a key individual difference factor that may shape how stress-related appraisals relate to emotional responses. Individuals with greater difficulties in emotion regulation may have limited access to adaptive strategies such as cognitive reappraisal, problem solving, or effective support seeking (Mansueto et al., 2024a,b). As a result, stress may be more likely to elicit sustained AS in these individuals. In contrast, individuals with better emotion regulation capacities may be more capable of buffering stress-related emotional reactions.

Consistent with this interpretation, the conditional indirect effect of perceived stress on EUD severity through AS severity was stronger at higher levels of emotion regulation difficulties. These findings suggest that emotion regulation difficulties may function as a vulnerability amplifier within the stress–AS severity–EUD pathway, rather than as an independent risk factor alone.

However, when the model was re-estimated in subgroups stratified by AS severity, this moderating effect did not reach statistical significance in either non-clinical or clinical anxiety subgroups. With a relatively small sample size (*n* = 96), the clinical anxiety subgroup likely had insufficient statistical power to detect such effects. Therefore, the moderating role of emotion regulation difficulties warrants cautious interpretation, particularly when generalized to participants with clinically elevated anxiety.

In the present study, our conceptual framework defines emotion regulation difficulties as a broad vulnerability trait rather than a cluster of independent mechanisms. Consistent with this conceptualization, we adopted DERS total score as a proxy for overall emotion regulation dysfunction. Notably, however, the DERS encompasses multiple distinct dimensions (e.g., impulse control difficulties, lack of emotional clarity), each reflecting a unique facet of regulatory processes. Thus, the moderating effect observed in this study may be primarily driven by specific components of emotion regulation difficulties, rather than by the global construct *per se*. Future research should examine whether individual DERS dimensions exert differential moderating effects on the stress–AS severity pathway. Such investigations would facilitate theoretical refinement and provide empirical support for the development of more targeted intervention strategies.

### 4.4 Sensitivity analysis

Sensitivity analyses utilizing recent craving scores as an alternative outcome yielded a similar pattern of associations. However, given the temporal mismatch between key variables (past-month stress, past-week AS severity, and 12-month EUD severity) and the cross-sectional study design, these results cannot be interpreted causally. While the findings confirm the robustness of our primary results, they do not allow for the establishment of temporal order among variables.

## 5 Strengths and limitations

Several strengths of the present study should be noted. To our knowledge, this is among the first studies to examine psychological mechanisms underlying EUD using a moderated mediation framework. By integrating perceived stress, AS severity, and emotion regulation difficulties, the study provides a theoretically informed model that may inform future research and intervention efforts. In addition, the relatively large clinical sample enhances the reliability of the observed associations.

Several limitations should also be acknowledged. First, while sensitivity analyses corroborated key findings and bolstered the robustness of our theoretical framework, the cross-sectional design precludes definitive inferences about causal relationships or temporal ordering in the associations among perceived stress, AS severity, and EUD severity. Longitudinal studies using a unified time frame are therefore warranted to clarify the directionality of the observed pathways. Second, all participants were male and recruited from a single rehabilitation center in Hunan Province, which may limit the generalizability of the findings to female populations or other regions. Third, self-report measures were used to assess perceived stress, AS severity, and emotion regulation, which may be subject to recall or response biases. Future studies incorporating multi-method assessments, such as behavioral or neurobiological measures, would strengthen the evidence base. Forth, as study inclusion required a positive hair test within the past six months, our sample may have overrepresented individuals with more persistent or severe patterns of etomidate use. Future studies should examine these associations in more heterogeneous samples. Finally, while age, educational attainment, family circumstances, and place of residence have been adjusted for as covariates, future studies should further consider additional potential confounding factors, such as the duration of heteropyrazine use. Furthermore, to yield more reliable norm-referenced data, future research may administer identical assessments concurrently to healthy control participants matched for region and age group.

## 6 Conclusion

In conclusion, the present study provides evidence that AS severity partially mediates the association between perceived stress and EUD severity, and that difficulties in emotion regulation amplify the stress–AS severity pathway. These findings contribute to a more nuanced understanding of stress-related emotional mechanisms in EUD. However, the boundary conditions underlying these associations remain unclear, and interpretations pertaining to self-medication or coping mechanisms should be advanced with caution. Clinically, interventions that target stress-related cognitive appraisals, anxiety symptom management, and emotion regulation skills may be particularly beneficial for individuals with EUD, especially those experiencing high levels of perceived stress. Specifically, strategies such as cognitive reappraisal can enhance emotion regulation capacity. When integrated with mindfulness-based interventions, these approaches enable individuals to achieve non-judgmental awareness of their emotional experiences. Furthermore, emotion-focused therapy and dialectical behavior therapy equip individuals with practical skills to manage intense emotional states more effectively, reduce impulsive behaviors, and foster emotional resilience.

## Data Availability

The raw data supporting the conclusions of this article will be made available by the authors, without undue reservation.
