# Supplementary material for: Perceived stress and severity of male etomidate use disorder in China: a moderated mediation model of anxiety symptoms severity and emotion regulation difficulties
Source: Front Psychol. 2026 May 20;17:1778758. doi: 10.3389/fpsyg.2026.1778758 (PMC13229857; doi:10.3389/fpsyg.2026.1778758)
Supplement: Supplementary file 1 [file Table_1.docx]

**Table 1 Missing Data Statistics for Study Variables**

| Variables | Missing (N) | Missing (%) |
| --- | --- | --- |
| Continuous Variables | 0 | 0.0 |
| Age | 0 | 0.0 |
| EUD Severity | 0 | 0.0 |
| AS Severity | 0 | 0.0 |
| Perceived Stress | 0 | 0.0 |
| DERS | 0 | 0.0 |
| VAS | 0 | 0.0 |
| Categorical Variables | 0 | 0.0 |
| Whether an only child | 0 | 0.0 |
| Whether a single-parent family | 1 | 0.2 |
| Residence | 0 | 0.0 |
| Middle school | 0 | 0.0 |
| High/Vocational school | 0 | 0.0 |
| Bachelor's degree or higher | 0 | 0.0 |

Note: N=556. DERS = Difficulties in Emotion Regulation. EUD = Etomidate Use Disorder. AS severity = Anxiety symptoms severity. VAS = Visual Analog Scale.

**Table 2 Table of Covariate Assignment.**

| Variables | Assignment/Encoding Methods |
| --- | --- |
| Age | Continuous variable |
| Whether an only child | Categorical variable |
| Yes | 1 |
| No | 0 |
| Whether a single-parent family | Categorical variable |
| Yes | 1 |
| No | 0 |
| Residence | Categorical variable |
| Rural | 0 |
| Urban | 1 |

**Table 3 Education Level Coding Method.**

| Education level | Middle school | High/ Vocational school | Bachelor's degree or higher |
| --- | --- | --- | --- |
| Primary school and below | 0 | 0 | 0 |
| Middle school | 1 | 0 | 0 |
| High/ Vocational school | 0 | 1 | 0 |
| Bachelor's degree or higher | 0 | 0 | 1 |

Note: Education level was recoded into three dummy variables, with primary school and below designated as the reference category. Each dummy variable was coded as 1 for participants belonging to the corresponding category, and 0 for all other participants.

**Table 4 The first-stage regression model.**

| Predictor | B | SE | t | p | 95% CI |
| --- | --- | --- | --- | --- | --- |
| constant | 0.622 | 0.258 | 2.41 | 0.016 | [0.117, 1.127] |
| Perceived Stress (X) | 0.268 | 0.040 | 6.66 | <0.001 | [0.189, 0.347] |
| DERS (W) | 0.316 | 0.041 | 7.70 | <0.001 | [0.235, 0.397] |
| X × W | 0.098 | 0.032 | 3.07 | 0.002 | [0.035, 0.161] |
| Age | -0.009 | 0.006 | -1.44 | 0.151 | [-0.021, 0.003] |
| Whether an only child | -0.046 | 0.083 | -0.56 | 0.576 | [-0.209, 0.117] |
| Whether a single-parent family | 0.019 | 0.080 | 0.24 | 0.813 | [-0.138, 0.176] |
| Residence | -0.517 | 0.199 | -2.59 | 0.010 | [-0.907, -0.127] |
| Middle school | -0.003 | 0.086 | -0.03 | 0.973 | [-0.172, 0.166] |
| High/Vocational school | 0.172 | 0.109 | 1.58 | 0.114 | [-0.041, 0.385] |
| Bachelor's degree or higher | 0.179 | 0.173 | 1.04 | 0.300 | [-0.160, 0.518] |

Note: Outcome: Anxiety Symptoms Severity; R² = 0.337, p < 0.001; ΔR² for interaction = 0.012, p = 0.002. All continuous variables were standardized prior to analysis.

**Table 5 The second-stage regression model.**

| Predictor | B | SE | t | p | 95% CI |
| --- | --- | --- | --- | --- | --- |
| constant | -0.544 | 0.289 | -1.880 | 0.061 | [-1.112, 0.025] |
| Perceived Stress (X) | \| 0.110 \| \| --- \| | 0.045 | 2.433 | 0.015 | [0.021, 0.200] |
| AS severity (M) | 0.303 | 0.045 | 6.746 | <0.001 | [0.215, 0.392] |
| Age | 0.014 | 0.007 | 2.00 | 0.046 | [0.000, 0.028] |
| Whether an only child | 0.057 | 0.094 | 0.60 | 0.547 | [-0.128, 0.242] |
| Whether a single-parent family | -0.055 | 0.092 | -0.59 | 0.553 | [-0.236, 0.126] |
| Residence | 0.201 | 0.229 | 0.88 | 0.379 | [-0.248, 0.650] |
| Middle school | -0.033 | 0.097 | -0.34 | 0.735 | [-0.224, 0.158] |
| High/Vocational school | -0.120 | 0.123 | -0.97 | 0.332 | [-0.362, 0.122] |
| Bachelor's degree or higher | 0.132 | 0.197 | 0.67 | 0.502 | [-0.254, 0.519] |

Note: Outcome: EUD severity; R² = 0.135, p < 0.001. All continuous variables were standardized prior to analysis.

**Table 6 The first-stage regression model by Non-clinical anxiety subgroup.**

| Predictor | B | SE | t | p | 95% CI |
| --- | --- | --- | --- | --- | --- |
| constant | 0.451 | 0.325 | 1.39 | 0.167 | [-0.189, 1.090] |
| Perceived Stress (X) | 0.299 | 0.047 | 6.423 | <0.001 | [0.208, 0.391] |
| DERS (W) | 0.197 | 0.047 | 4.21 | <0.001 | [0.105, 0.288] |
| X × W | -0.032 | 0.043 | -0.74 | 0.461 | [-0.117, 0.053] |
| Age | -0.002 | 0.007 | -0.25 | 0.799 | [-0.016, 0.012] |
| Whether an only child | -0.066 | 0.101 | -0.65 | 0.514 | [-0.265, 0.133] |
| Whether a single-parent family | -0.030 | 0.098 | -0.30 | 0.761 | [-0.223, 0.163] |
| Residence | -0.469 | 0.271 | -1.73 | 0.084 | [-1.002, 0.064] |
| Middle school | 0.093 | 0.107 | 0.87 | 0.384 | [-0.117, 0.304] |
| High/Vocational school | 0.104 | 0.132 | 0.78 | 0.433 | [-0.157, 0.364] |
| Bachelor's degree or higher | 0.263 | 0.209 | 1.26 | 0.209 | [-0.148, 0.674] |

Note: N = 460; Outcome: Anxiety Symptoms Severity; R² = 0.171, p < 0.001; ΔR² for interaction = 0.001, p = 0.461; Index of moderated mediation = -0.008, BootSE = 0.010, 95% CI [-0.028, 0.011]. All continuous variables were standardized prior to analysis.

**Table 7 The second-stage regression model by Non-clinical anxiety subgroup.**

| Predictor | B | SE | t | p | 95% CI |
| --- | --- | --- | --- | --- | --- |
| constant | -0.900 | 0.335 | -2.69 | 0.008 | [-1.558, -0.242] |
| Perceived Stress (X) | 0.085 | 0.048 | 1.76 | 0.080 | [-0.010, 0.180] |
| AS severity (M) | 0.258 | 0.048 | 5.40 | <0.001 | [0.164, 0.352] |
| Age | 0.016 | 0.007 | 2.14 | 0.033 | [0.001, 0.030] |
| Whether an only child | 0.009 | 0.104 | 0.08 | 0.932 | [-0.196, 0.214] |
| Whether a single-parent family | -0.085 | 0.101 | -0.83 | 0.405 | [-0.284, 0.115] |
| Residence | 0.455 | 0.280 | 1.63 | 0.105 | [-0.095, 1.006] |
| Middle school | 0.206 | 0.110 | 1.86 | 0.063 | [-0.011, 0.422] |
| High/Vocational school | 0.081 | 0.137 | 0.59 | 0.552 | [-0.187, 0.350] |
| Bachelor's degree or higher | 0.067 | 0.215 | 0.31 | 0.757 | [-0.357, 0.491] |

Note: N = 460; Outcome: EUD Severity; R² = 0.108, p < 0.001. All continuous variables were standardized prior to analysis.

**Table 8 The first-stage regression model by Clinical anxiety subgroup.**

| Predictor | B | SE | t | p | 95% CI |
| --- | --- | --- | --- | --- | --- |
| constant | -0.715 | 0.656 | -1.09 | 0.279 | [-2.019, 0.589] |
| Perceived Stress (X) | 0.074 | 0.100 | 0.74 | 0.462 | [-0.125, 0.274] |
| DERS (W) | 0.530 | 0.100 | 5.33 | <0.001 | [0.333, 0.729] |
| X × W | -0.087 | 0.099 | -0.88 | 0.379 | [-0.284, 0.109] |
| Age | 0.025 | 0.021 | 1.18 | 0.243 | [-0.017, 0.067] |
| Whether an only child | 0.182 | 0.208 | 0.88 | 0.383 | [-0.231, 0.596] |
| Whether a single-parent family | 0.738 | 0.219 | 3.37 | 0.001 | [0.302, 1.174] |
| Residence | 0.089 | 0.386 | 0.23 | 0.818 | [-0.678, 0.856] |
| Middle school | -0.149 | 0.199 | -0.75 | 0.455 | [-0.546, 0.247] |
| High/Vocational school | 0.399 | 0.268 | 1.49 | 0.140 | [-0.134, 0.933] |
| Bachelor's degree or higher | -1.327 | 0.447 | -2.97 | 0.004 | [-2.215, -0.439] |

Note: N = 96; Outcome: Anxiety Symptoms Severity; R² = 0.390, p < 0.001; ΔR² for interaction = 0.006, p = 0.379; Index of moderated mediation = -0.017, BootSE = 0.017, 95% CI [-0.049, 0.020]. All continuous variables were standardized prior to analysis.

**Table 9 The second-stage regression model by Clinical anxiety subgroup.**

| Predictor | B | SE | t | p | 95% CI |
| --- | --- | --- | --- | --- | --- |
| constant | 0.630 | 0.773 | 0.81 | 0.417 | [-0.907, 2.167] |
| Perceived Stress (X) | 0.046 | 0.111 | 0.41 | 0.681 | [-0.174, 0.266] |
| AS severity (M) | 0.133 | 0.112 | 1.18 | 0.240 | [-0.090, 0.355] |
| Age | -0.005 | 0.025 | -0.19 | 0.847 | [-0.054, 0.045] |
| Whether an only child | 0.403 | 0.247 | 1.63 | 0.107 | [-0.088, 0.894] |
| Whether a single-parent family | 0.173 | 0.268 | 0.64 | 0.521 | [-0.361, 0.707] |
| Residence | -0.768 | 0.451 | -1.70 | 0.092 | [-1.665, 0.129] |
| Middle school | 0.144 | 0.236 | 0.61 | 0.541 | [-0.324, 0.613] |
| High/Vocational school | -0.241 | 0.323 | -0.75 | 0.457 | [-0.884, 0.401] |
| Bachelor's degree or higher | 0.414 | 0.539 | 0.77 | 0.445 | [-0.658, 1.486] |

Note: N = 96; Outcome: EUD severity; R² = 0.123, p = 0.231. All continuous variables were standardized prior to analysis.
